# Human Papillomavirus and Risk of Colorectal Cancer: An Analysis of Nationwide Claims Data

**DOI:** 10.3390/medicina58101461

**Published:** 2022-10-15

**Authors:** Chih-Hsiung Hsu, Yu-Jyun Lin, Yong-Chen Chen, I-Lan Liu, San-Lin You, Je-Ming Hu, Tzu-Chiao Lin, Pi-Kai Chang, Chao-Yang Chen, Yu-Ching Chou, Chien-An Sun

**Affiliations:** 1 School of Public Health, National Defense Medical Center, Taipei City 114, Taiwan; 2 Department of Medicine, College of Medicine, Fu-Jen Catholic University, New Taipei City 242, Taiwan; 3 Data Science Center, College of Medicine, Fu-Jen Catholic University, New Taipei City 242, Taiwan; 4 Graduate Institute of Medical Sciences, National Defense Medical Center, Taipei City 114, Taiwan; 5 Division of Colorectal Surgery, Department of Surgery, Tri-Service General Hospital, National Defense Medical Center, Taipei City 114, Taiwan; 6 Department of Public Health, College of Medicine, Fu-Jen Catholic University, New Taipei City 242, Taiwan

**Keywords:** cohort study, colorectal cancer, human papillomavirus, medical claims dataset

## Abstract

*Background and Objectives*: Although human papillomavirus (HPV) is a major etiology of cervical and anogenital cancers, whether it is associated with colorectal carcinogenesis is yet undetermined. *Materials and Methods*: The longitudinal association of HPV infection with colorectal cancer (CRC) was evaluated using 2000–2013 data from a nationwide Taiwanese claims database. In this retrospective cohort study, 358 patients with primary HPV diagnoses (HPV-infected cohort) and 1432 patients without such a diagnosis (HPV-uninfected cohort) were recruited between 2000 and 2006. Both cohorts were followed up to identify CRC incidences from 2006 to 2013. Hazard ratios (HRs) and their 95% confidence intervals (CIs) derived from Cox proportional hazards models were used to estimate the association between HPV and CRC risk. *Results*: The HPV-infected cohort had a significantly higher cumulative incidence of CRC than the HPV-uninfected cohort. The presence of HPV was associated with an increased risk of CRC (adjusted HR, 1.63; 95% CI, 1.02–3.62). Furthermore, the significant HPV–CRC risk association was evident in both sexes. *Conclusions*: This population-based cohort study reveals longitudinal evidence that HPV is associated with an increased risk of CRC. Further studies are required to verify the role of HPV in colorectal carcinogenesis.

## 1. Introduction

Colorectal cancer (CRC) is the third and second leading cause of cancer in men and women, respectively, based on the GLOBOCAN estimates of worldwide cancer incidence rates [1]. The incidence rates of CRC are rising rapidly in Asia, and the incidence rates of CRC in many Asian countries are similar to those in the western ones [2]. CRC is associated with lifestyle and inherited genetic factors [3]. In 2018, about 2.2 million cancer cases, or 13% of global cancer incidence rates, were attributable to infection [4]. The literature also suggests a link between CRC risk and infectious agents, especially human papillomavirus (HPV) [5,6,7]. 

A non-enveloped circular, double-stranded DNA virus, HPV, is the major cause of cervical cancer [8]. Similarly, the HPV–CRC cancer association was first reported by Kirgan et al. in 1990 [9], and HPV infection in relation to colorectal carcinogenesis has since been evaluated by numerous research groups [10,11,12,13,14,15,16,17,18,19,20,21,22,23,24,25,26,27,28,29,30,31,32]. The possible association between CRC and HPV, however, remains controversial. Numerous studies have suggested an association of HPV infection with the development of CRC [10,11,12,13,14,15,16,18,22,24,27,28,30,31], but several investigations have indicated no evidence of this link [17,20,21,23,25,26,29,32]. The appropriate care for CRC and prevention of this disease would be strongly influenced by such a relationship. Therefore, we employed claims data from Taiwan’s nationwide National Health Insurance Research Database (NHIRD) to assess the association between HPV and CRC.

## 2. Methods

### 2.1. Data Source

We used retrospective data from the Taiwan NHIRD, which comprises medical claims data from the National Health Insurance (NHI) program. The NHI is a single-payer, universal, and compulsory healthcare program in which more than 98% of the 23 million residents of Taiwan are enrolled [33]. The NHIRD contains demographic data of insurants; the clinical data on drug prescriptions, procedures, surgeries, and diagnostic codes in the format of the International Classification of Disease, Ninth Revision, Clinical Modification (ICD-9-CM); and the use of inpatient and outpatient care facilities. The NHIRD has been used for high-quality epidemiological research [34,35], and the information on diagnoses, drug prescriptions, and hospitalizations has been shown to be of good validity [36,37,38]. We used data from the Longitudinal Health Insurance Database (LHID), which covers a cohort of 2 million NHI beneficiaries sampled at random from the NHIRD’s registry for the period of 1 January 2000 to 31 December 2013. The individuals in the NHIRD cohort and those in the LHID cohort do not significantly differ in terms of sex, age, or healthcare costs [33]. The data provided to researchers are entirely anonymous because of de-identification through the scrambling of the beneficiaries’ ID codes. Written or verbal patient consent was thus waived. The Institutional Review Board of Fu-Jen Catholic University (FJUIRB No: C104014, approval date: 2015-10-06) approved this study’s protocol. 

### 2.2. Study Population

**Figure 1:**
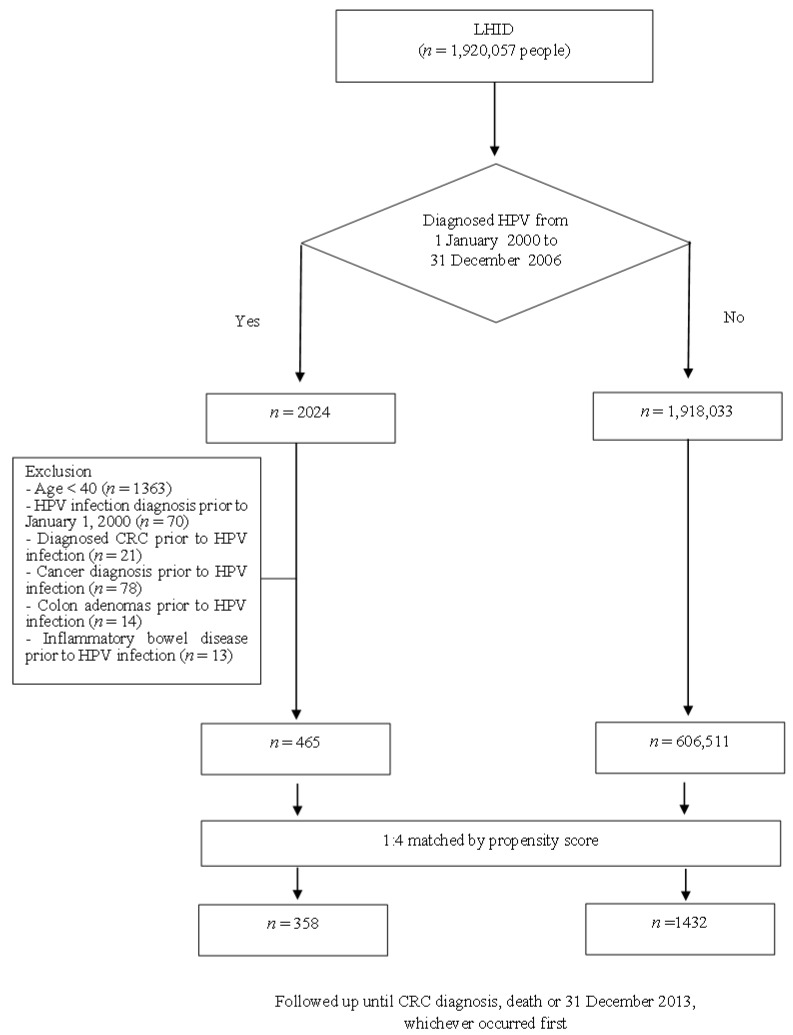
Study flowchart. LHID, Longitudinal Health Insurance Database; HPV, human papillomavirus; CRC, colorectal cancer; CCI, Charlson comorbidity index.

All patient data analyzed in the present study were abstracted from the LHID. Patients with an HPV diagnosis between 1 January 2000 and 31 December 2006 were identified on the basis of ICD-9-CM codes [39] 079.4, 078.11, 795.05, 795.09, 795.15, 795.19, 796.75, and 796.79. Initially, we enrolled 2024 individuals who had new HPV diagnoses (the HPV cohort). Given that people aged more than 40 years were considered at-risk for CRC in Taiwan [40], we excluded patients aged younger than 40 years (*n* = 1362). In addition, patients with diagnosed HPV infection prior to 1 January 2000 (*n* = 70); patients with a cancer diagnosis (ICD-9-CM codes 140–209, and 235–239; *n* = 65), a colon adenomas diagnosis (ICD-9-CM code 211, *n* = 9), or an inflammatory bowel disease diagnosis (ICD-9-CM codes 555 and 556, *n* = 12); and patients with a CRC diagnosis prior to HPV infection (*n* = 21) were also excluded. Finally, 465 patients with a new HPV diagnosis were included in this study as the HPV-infected cohort. To ensure the accurate identification of patients with HPV, we enrolled only those patients who had two or more outpatient visits with HPV diagnostic codes. The date of HPV diagnosis was assigned as the index date. To recruit an HPV-uninfected cohort, we randomly selected individuals aged 40 years or older without an HPV diagnosis between 1 January 2000 and 31 December 2006. Patients in the HPV-uninfected cohort were assigned a reference date corresponding to the index date of the matched patients in the HPV-infected cohort. To control for differences in underlying conditions between the two cohorts, we applied propensity score matching for the HPV-infected cohort to the matched HPV-uninfected cohort. The propensity score was calculated for each patient using a logistic regression model with covariates of age, sex, comorbidities, and the frequency of outpatient visits. The matching ratio was 1:4 for the HPV-infected to HPV-uninfected cohorts for the consideration of increasing the power and efficiency of statistical analyses [41]. Finally, 358 patients with HPV infection and 1432 patients without HPV infection were included in the analysis. Follow-up was initiated from the index date until 31 December 2013, death (based on withdrawal from the NHI), or a first primary diagnosis of CRC, whichever occurred first (Figure 1). For the HPV-infected and HPV-uninfected cohorts, the mean follow-up period was 6.8 (±2.1) and 7.4 (±1.6) years, respectively.

### 2.3. CRC Identification

The main outcome was the incidence of CRC, identified on the basis of a primary diagnosis with one of the following ICD-9-CM codes: 153, 153.0, 153.1, 153.2, 153.3, 153.4, 153.5, 153.6, 153.7, 153.8, 153.9, 154, 154.0, 154.1, 154.2, 154.3, and 154.8. We consulted the Registry for Catastrophic Illness Patient Database (RCIPD), which is an NHIRD subset, to ensure that the identifications were sufficiently accurate. We can thoroughly and accurately track the incident CRC cases using data in the RCIPD [38]. In Taiwan, a patient with cancer is not given a certificate of catastrophic illness unless they have an official hospital-issued certificate of the diagnosis that is supported by laboratory results, diagnostic imaging, or histological findings. 

### 2.4. Covariate Assessment and Adjustment

The covariates of interest were patient demographics, comorbidities, general health, and healthcare utilization. The following comorbidities were identified from inpatient and outpatient files: diabetes mellitus (ICD-9-CM code 250), hypertension (ICD-9-CM codes 401, 402, 403, 404, and 405), chronic obstructive pulmonary disease (COPD) (ICD-9-CM codes 491, 492, and 496), hypercholesterolemia (ICD-9-CM codes 272.0, 272.1, 272.2, and 272.4), and alcohol-related conditions (ICD-9-CM codes 571.0, 571.1, 571.2, and 571.3 for alcoholic liver disease, 303 for alcohol dependence, 305.0 for alcohol abuse, and 291 for alcohol-induced mental disorders). Comorbidities were defined in a patient if he or she was diagnosed with any of the aforementioned diseases on at least two or more outpatient claims or one or more inpatient claims in the study period. In the present study, hypertension, diabetes mellitus, or hypercholesterolemia was used as a surrogate measure of obesity. COPD was considered a proxy indicator of smoking, and alcohol-related conditions were utilized as a substitute variable for alcohol intake. In addition, general health status was indicated by a patient’s CCI score. In this scoring system, the presence of major concomitant diseases is summed, with each disease appropriately weighted; previous studies have validated the use of the CCI with administrative databases containing ICD-9-CM codes [42]. Furthermore, healthcare utilization was reflected by the frequency of outpatient visits. These covariates were included in the regression models for adjustment.

### 2.5. Statistical Analysis

We used the chi-squared or Student t-test to determine between cohort differences in the baseline comorbidities and demographic characteristics. Cumulative CRC incidence was evaluated using a Kaplan–Meier curve, and the cumulative CRC risks in the cohorts were compared through the log-rank test. Hazard ratios (HRs) and 95% confidence intervals (CIs) derived from the multivariable Cox proportional hazards models were used to assess HPV–CRC risk association. We evaluated the proportionality assumption of Cox models using the log minus log plot of survival [43]. Statistical significance was considered to be indicated by a two-tailed *p* < 0.05. We performed all analyses in SAS version 9.4 (SAS Institute, Cary, NC, USA).

## 3. Results

The characteristics of the study cohorts are presented in Table 1. The mean age (± standard deviation) of the HPV-infected and uninfected cohorts was 51.9 (±10.1) and 51.8 (±10.0) years, respectively. There were no significant differences in the distributions of age, sex, CCI score, frequency of outpatient visits, and comorbidities between the HPV-infected cohort and the HPV-uninfected cohort due to the propensity score matching schemes.

**Figure 2:**
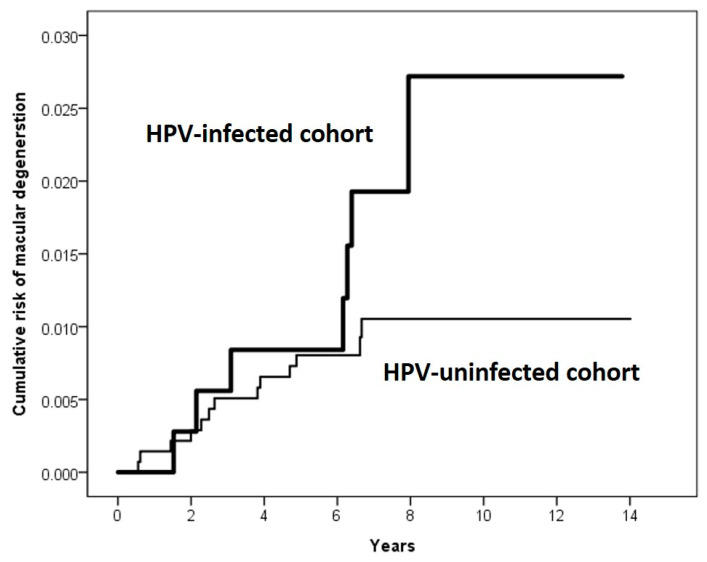
Kaplan–Meier analysis of the cumulative incidence of colorectal cancer in the HPV-infected cohort and the HPV-uninfected cohort.

The mean follow-up period was 6.9 years. During the follow-up, 7 patients were identified as incident CRC in the HPV-infected cohort and 13 were noted in the HPV-uninfected cohort for incidence rates of 26.48 and 14.17 per 10,000 person-years, respectively. The HPV-infected cohort had a significantly greater risk of CRC in comparison with the HPV-uninfected cohort, with an adjusted HR of 1.63 (95% CI, 1.02–3.62) (Table 2). Of note, the significant HPV–CRC risk association was evident in both sexes (Table 2). The Kaplan–Meier curves for the cumulative incidence of CRC among the two cohorts are displayed in Figure 2. The cumulative incidence of CRC is significantly higher in the HPV-infected cohort than in the HPV-uninfected cohort (*p* = 0.036).

A differential pattern of association between HPV infection and CRC risk was demonstrated according to the frequency of clinical visits. Relative to patients without HPV infection, a higher HR was noted among patients having the highest frequency of clinical visits for a diagnosed HPV infection (adjusted HR, 2.03; 95% CI, 1.02–7.18). A trend, with a significant increase in HR with increasing frequency of clinical visits for a diagnosed HPV infection, was noted (*p* = 0.004) (Table 3).

## 4. Discussion

Hereditary and environmental factors contribute to the development of CRC and, thus, affect CRC risk. In particular, the greatest increase in risk is based on inherited susceptibility [44]. If this cancer were found to have an infectious etiology, the potential for diagnostic tools and therapeutic interventions would be substantial. In the present large-scale population-based cohort study, we documented a significant association of HPV with increased CRC risk in both sexes. 

HPV is widely recognized to play an important role in the development of some carcinoma types, including cervical cancer, which is positive for HPV in 90% of cases, and head and neck tumors, for which the percentage is approximately 30% [45,46]. Although how HPV precisely affects CRC risk has been the topic of numerous studies, an association between HPV and CRC is controversial, with several publications suggesting that HPV infection of the colonic mucosa may contribute to the development of CRC [10,11,12,13,14,15,16,18,22,24,27,28,30,31]. However, there have been several investigations that failed to establish a link between HPV and CRC [17,20,21,23,25,26,29,32]. These discrepant findings could result from geographic differences in the study populations, regional differences in HPV prevalence, or variations in methods of HPV detection and choice of the material for analysis (fresh/paraffin). The findings of this study support the findings of investigations reporting that the risk of CRC is higher in individuals with HPV [10,11,12,13,14,15,16,18,22,24,27,28,30,31]. Zhang et al. presented evidence that, in the Chinese population, HPV infection is related to CRC risk [31]. In addition, in their HPV-type-specific polymerase chain reaction (PCR) analysis of tissues from Taiwanese patients, Cheng et al. determined that 29% of adenomas and 53% of colorectal carcinomas contained HPV DNA [47]. Indeed, several studies have pointed out that high-risk HPVs are present in human CRCs, specifically types 16, 18, 31, 33, and 35 [19,24,48,49]. Furthermore, several meta-analyses have indicated associations of high-risk HPV types 16, 18, 31, 33, and 35 with CRC risk [6,19,24]. It is well known that high-risk HPV early proteins, including E5, E6, and E7 oncoproteins, increase cellular alteration and probably lead to HPV-induced carcinogenesis [50,51,52]. The high prevalence of infection in colorectal tumors with a high-risk HPV type (HPV-16) and evidence of viral genome integration [19,24,27] support the possibility that HPV may have a role in colorectal carcinogenesis. Collectively, these data quantitatively support the notion that HPV infection is associated with an elevated risk of CRC.

We were able to analyze an unselected study population in a real-life setting because we employed data from large and representative population-based NHI cohorts; this is a strength of the study. A further strength is that we employed routine database records and could thus minimize selection and nonresponse biases. However, the limitations of this study must be considered in the interpretation of the results. Data on potential confounders are often lacking from insurance claims data [53], and this could have affected the present study. Information on the virus types of HPV or variables related to lifestyle, such as alcohol consumption and cigarette smoking, were not available in the studied claims dataset. Furthermore, data on sexual behaviors, HPV vaccination, and body mass index were also not factored in the claims database. Thus, we were unable to account for these factors in our models, and it is likely that our findings could be subject to residual confounding. Of note, HPV was significantly associated with cervical cancer and head and neck cancers [45,46]. Thus, ignoring competing events in the analysis of HPV–CRC association may lead to biased results. Overall, although we did not have data on the types of HPV, Cheng et al. found HPV DNA, especially type 16, in 29.7% and 52.9% of adenomas and carcinomas, respectively, after PCR and Southern blot hybridization of colorectal tumors from Taiwanese patients [47]. These findings implicate HPV DNA in the development of colorectal carcinomas in the context of Taiwanese patients. 

## 5. Conclusions

In conclusion, despite the limitations described above, our study provides longitudinal evidence that HPV infection is associated with an increased risk of developing CRC in the Taiwanese population. It should be pointed out, however, that this observation needs to be substantiated by further studies to elucidate the role of HPV in colorectal carcinogenesis. 

## Figures and Tables

**Table 1:** Baseline demographics and comorbidities in the HPV-infected and HPV-uninfected cohorts.

Variable	HPV-Infected Cohort *n* = 358	HPV-Uninfected Cohort *n* = 1432	*p*-Value
Age, years (mean (SD))	51.9 (10.8)	51.8 (10.0)	0.817
CCI (mean (SD))	0.3 (0.7)	0.3 (1.1)	0.914
No. of annual outpatient visits (mean (SD))	15.3 (37.0)	12.2 (57.6)	0.513
Sex (No. (%))			0.857
Female	146 (40.8)	573 (40.2)	
Male	212 (59.2)	857 (59.8)	
Comorbidities (No. (%))			
COPD	78 (21.8)	275 (19.2)	0.267
Hypertension	150 (41.9)	526 (36.7)	0.092
Diabetes mellitus	81 (22.6)	316 (22.1)	0.831
Hypercholesterolemia	120 (33.5)	426 (29.8)	0.178
Alcoholic liver disease	8 (2.2)	25 (1.8)	0.513
Alcohol dependence	6 (1.7)	23 (1.6)	0.987
Alcohol abuse	2 (0.6)	10 (0.7)	0.976
Alcohol-induced mental disorders	3 (0.8)	8 (0.6)	0.768

CCI, Charlson comorbidity index; COPD, chronic obstructive pulmonary.

**Table 2:** Incidence rates of colorectal cancer (CRC) in study cohorts and multivariable Cox proportional hazards regression model analysis for the association between HPV infection and the risk of CRC.

Variable	HPV-Infected Cohort			HPV-Uninfected Cohort			Adjusted HR (95% CI)
	No. of CRC Cases	PYs	Incidence Rate (per 10,000)	No. of CRC Cases	PYs	Incidence Rate (per 10,000)	
Total	7	2644	26.48	13	9177	14.17	1.63 (1.02–3.62)
Sex							
Female	3	1096	27.37	5	4003	12.49	1.91 (1.09–6.36)
Male	4	1548	25.84	8	5709	14.01	1.54 (1.08–4.01)

PYs: person-years; HR: hazard ratio; CI: confidence interval. Hazard ratios for the whole group analysis were adjusted for age, sex, the Charlson comorbidity index, comorbidities of chronic obstructive pulmonary disease, hypertension, diabetes mellitus, hypercholesterolemia, alcohol-related conditions, number of annual outpatient visits, and the index date. Hazard ratios for the sex-specific analyses were adjusted for age, the Charlson comorbidity index, comorbidities of chronic obstructive pulmonary disease, hypertension, diabetes mellitus, hypercholesterolemia, alcohol-related conditions, number of annual outpatient visits, and the index date.

**Table 3:** Multivariable Cox proportional hazards regression model analysis for the association between the risk of colorectal cancer (CRC) associated with frequency of clinical visits for a diagnosed HPV infection.

Frequency of Clinical Visits for Diagnosed HPV Infection	No. of CRC Cases	PYs	Incidence Rate (per 10,000)	Adjusted HR (95% CI)
HPV-uninfected cohort	13	9711	10.17	1.00 (reference)
1–2	4	1599	23.00	1.81 (0.59–5.57)
>2	3	1045	49.34	2.03 (1.02–7.18)
*p* for trend				0.004

PYs: person-years; HR, hazard ratio; CI, confidence interval. Hazard ratios were adjusted for age, sex, the Charlson comorbidity index, comorbidities of chronic obstructive pulmonary disease, hypertension, diabetes mellitus, hypercholesterolemia, alcohol-related conditions, number of annual outpatient visits, and the index date.

## Data Availability

No additional data are available.
